# Does Cognitive Self-Report Measure Type Differentially Predict Cognitive Decline? A Systematic Review

**DOI:** 10.1093/geroni/igaa057.936

**Published:** 2020-12-16

**Authors:** Rachel Wion, Nikki Hill, Tyler Bell, Jacqueline Mogle, Jennifer Yates, Iris Bhang

**Affiliations:** 1 Indiana University, Indianapolis, Indiana, United States; 2 Pennsylvania State University, University Park, Pennsylvania, United States; 3 University of Nottingham, Nottingham, United Arab Emirates

## Abstract

Up to 47% of older adults without measurable cognitive impairment report difficulties with memory and thinking which potentially increases their risk for developing cognitive decline. Many measures are used for assessing self-reported cognition; however, certain types of these measures may be more predictive of cognitive decline. The purpose of this systematic review was to compare the role of cognitive self-report measure types in predicting risk for cognitive decline. PubMed, CINAHL, and PsycINFO databases were searched using the following inclusion criteria: longitudinal studies, outcome of cognitive decline, and two or more cognitive self-report measures. A total of 4,319 articles were identified during the initial search and narrowed to 19 final articles. The Quality in Prognosis Studies tool was used to determine study quality. Six comparison themes emerged during synthesis: self-reported cognition or memory with or without worry; self-reported global cognition or self-reported memory; self-reported memory decline and self-reported executive function decline; self-reported cognition and self-reported memory by others; self-reported memory and self-reported memory problems in comparison with peers; and self-reported memory and self-reported memory affecting daily function. Self-reported memory decline with worry and self-reported memory problems by others were most predictive of future impairment. It was difficult to definitively determine whether certain cognitive self-report measure types where more predictive of risk for cognitive decline because there were very few articles in some of the comparison groups. Future investigations of self-reported cognition should focus on using measures that have been shown to be the most efficacious at predicting risk for cognitive decline.

